# Cathepsin L-induced galectin-1 may act as a proangiogenic factor in the metastasis of high-grade serous carcinoma

**DOI:** 10.1186/s12967-019-1963-7

**Published:** 2019-07-03

**Authors:** Md Zahidul I. Pranjol, Dmitry A. Zinovkin, Annelie R. T. Maskell, Laura J. Stephens, Sergey L. Achinovich, Dmitry M. Los’, Eldar A. Nadyrov, Michael Hannemann, Nicholas J. Gutowski, Jacqueline L. Whatmore

**Affiliations:** 1 Institute of Biomedical and Clinical Science, University of Exeter Medical School, Exeter, Devon EX1 2LU UK; 2 William Harvey Research Institute, Barts and the London School of Medicine and Dentistry, Queen Mary University of London, London, EC1M 6BQ UK; 3 Department of Pathology, Gomel State Medical University, 246000 Gomel, Belarus; 4 Department of Anatomical Pathology, Gomel Regional Clinical Oncological Dispensary, 246012 Gomel, Belarus; 5 Gomel Regional Clinical Oncological Dispensary, 246012 Gomel, Belarus; 6 Royal Devon and Exeter NHS Foundation Trust, Exeter, Devon EX2 5DW UK

**Keywords:** Galectin-1, Angiogenesis, Metastasis, High-grade serous carcinoma, Signalling pathways

## Abstract

**Background:**

New treatment options for metastasised high-grade serous carcinoma (HGSC) are urgently needed. HGSC frequently metastasises to the omentum, inducing angiogenesis in the local omental microvasculature to facilitate tumour growth. We previously showed that HGSC-secreted cathepsin L (CathL) induces pro-angiogenic changes in disease relevant human omental microvascular endothelial cells (HOMECs), suggesting a role in tumour angiogenesis. Here we investigate whether CathL acts by inducing local production of the carbohydrate-binding protein galectin-1 (Gal1), which has been reported to be involved in tumourigenesis in other tumours.

**Methods:**

HOMECs were used for all experiments. Gal1 mRNA and protein levels were measured by RT-PCR and ELISA respectively. Gal1-induced cell proliferation was assessed using WST-1 assay, migration using a transwell assay and in vivo Gal1 expression by immunohistochemistry.

**Results:**

CathL transcriptionally regulated HOMEC production and secretion of Gal1 via activation of NFκB (significantly inhibited by sulfasalazine). Gal1 significantly enhanced HOMEC migration (p < 0.001) and proliferation (p < 0.001), suggesting an autocrine action. The latter was significantly reduced by the MEK/ERK1/2 inhibitors U0126 and PD98059 suggesting downstream activation of this pathway. Immunohistochemical analysis of omenta from HGSC patients with or without metastatic disease demonstrated a positive correlation between Gal1 expression and number of microvessels (r = 0.8702, p < 0.001), and area of vessels (r = 0.7283, p < 0.001), supporting a proangiogenic role for Gal1 in omental metastases.

**Conclusion:**

HOMEC Gal1 transcription and release in response to CathL secreted from metastasising HGSC acts in an autocrine manner on the local microvasculature to induce pro-angiogenic changes, highlighting a potential new therapeutic target.

**Electronic supplementary material:**

The online version of this article (10.1186/s12967-019-1963-7) contains supplementary material, which is available to authorized users.

## Background

Effective treatment of patients suffering from advanced, high-grade serous carcinoma (HGSC) is still clinically challenging [1], with development of new therapies impeded by our limited understanding of the cellular mechanisms involved in HGSC metastasis.

Primary HGSC cells commonly metastasise to the omentum where secondary tumour establishment requires local neoangiogenesis to supply oxygen and nutrients. This process involves activation of a proangiogenic phenotype in the local microvascular endothelial cells (ECs), facilitated by complex cross-talk between the host omental cells and tumour cells. Growth factors and chemokines are locally secreted into the tumour microenvironment and may present potential therapeutic targets. For instance, vascular endothelial growth factor (VEGF) is highly expressed and over-secreted in HGSC [2] and thus, several anti-VEGF based therapies e.g. bevacizumab, have been developed. However, the observation that these therapies have only limited benefit in advanced disease [3–7], and that non-VEGF pathways may play a role in HGSC metastasis to the omentum has raised the possibility that alternative pro-angiogenic factors may be important e.g. cathepsins D (CathD) and L (CathL) [8–11]. It is thus important to understand the mechanisms by which these proteins activate angiogenic pathways in metastatic HGSC.

Our preliminary data suggested that CathL induces a differential expression of galectin1 (Gal1) mRNA (LGALS1) in disease-relevant human omental microvascular endothelial cells (HOMECs). Gal1, a carbohydrate-binding protein is reported to have a range of cellular effects [12]. In cancer, increased Gal1 expression is linked to tumour progression, possibly contributing to cancer cell invasion and metastasis formation [13, 14], and is associated with increased rates of disease reoccurrence [15–19], including in HGSC [20]. Gal1 may also play a role in cancer angiogenesis since Gal1 knockout mice had severely impeded vessel formation and disrupted tumour growth [21]. This is supported by the observation that, in vitro, exogenous Gal1 induced angiogenic tube-formation in non-disease EC models such as human umbilical vein ECs (HUVECs) and EA.hy926 [22, 23]. Despite this emerging role for Gal1 in tumour angiogenesis, the molecular mechanisms involved in its cellular effects in ECs remain poorly understood.

In this investigation, we examined the role and potential cellular regulation of Gal1 in metastasis of HGSC to the omentum, using both in vitro studies on disease-relevant HOMECs and immunohistochemical studies of patient samples. We show, for the first time that CathL, known to be secreted from HGSC, induces secretion of Gal1 from HOMECs in a transcriptionally regulated manner. Gal1 then acts in an autocrine manner to induce a proangiogenic phenotype, i.e. enhanced proliferation and migration, in these cells, via activation of the intracellular kinases ERK1/2 and AKT. Finally, we report, for the first time, an increased in vivo omental endothelial expression of Gal1 that correlates with increased microvessel density and vessel area in omentum of patients with serous carcinoma (with HGSC metastasis) compared with normal omentum or omentum of patients with non-metastatic serous carcinoma, indicating a potential proangiogenic role for Gal1 that confirms our in vitro observation. These combined data strongly indicate Gal1 as a proangiogenic factor in advanced HGSC that could be targeted therapeutically.

## Materials and methods

### Primary HOMEC isolation and culture

Non-malignant omental tissue samples were collected from patients undergoing surgery at the Royal Devon and Exeter NHS Foundation Trust (Exeter, United Kingdom) with ethical approval and informed written consent. HOMECs were isolated using collagen-digestion and anti-CD31 magnetic beads, characterised and cultured as primary cells as previously described [24]. Briefly, HOMECs were cultured in endothelial cell (EC) growth media (MV2, PromoCell, Heidelberg, Germany) supplemented with supplied growth factors, 5% (v/v) foetal calf serum (FCS) and 0.1% (v/v) gentamycin (Sigma, Poole, UK). Cells were maintained at 37 °C in a humidified atmosphere supplemented with 5% CO_2_.

### Cell based ELISA

Phosphorylation levels of NFκB, ERK1/2 and AKT were measured using specific cell-based ELISA kits (Bio-Techne Ltd., Abingdon, UK) according to the manufacturer’s instructions. Cells were starved, and subsequently treated ± recombinant human VEGF165 (20 ng/ml, positive control; Peprotech, London, UK), CathL (50 ng/ml; from human liver; Sigma-Aldrich, Poole, UK) and recombinant human Gal1 (50 ng/ml; Sigma-Aldrich, Poole, UK) ± NFκB, ERK1/2 and AKT inhibitors at their given concentrations (Table 1) for 4, 10 min or 4 h. To confirm the effect of these inhibitors, HOMECs were pre-incubated with the inhibitors and then co-treated ± proangiogenic factors. Fluorescence intensity was measured, and the results were expressed as fold change in phospho-NFκB (p65), -ERK1/2 or -AKT relative to total NFκB, ERK or AKT levels (compared to control).

**Table 1:** Concentrations of treatments added

Treatments	Purpose	Pre-incubation time	Concentration(s)	Source
VEGF165	Positive control	n/a	20 ng/ml	Peprotech (London, UK)
Tumour necrosis factor-alpha (TNF-α)	Positive control	n/a	160 pg/ml	Enzo Life sciences, (Exeter, UK)
CathL	Treatment	n/a	50 ng/ml	Sigma-Aldrich (Poole, UK)
Gal1	Treatment	n/a	1, 5, 10, 25, 50, 125 ng/ml	Sigma-Aldrich (Poole, UK)
Sulfasalazine	NFκB inhibitor	24 h	100 µmol/l	Stratech (Suffolk, UK)
U0126	MEK/ERK1/2 inhibitor	20–30 min	10 µmol/l	Stratech (Suffolk, UK)
PD98059	MEK/ERK1/2 inhibitor	20–30 min	25 µmol/l	Stratech (Suffolk, UK)
LY294002	PI3K inhibitor	1–2 h	25 µmol/l	Stratech (Suffolk, UK)
MK2206	AKT inhibitor	1–2 h	5 µmol/l	Stratech (Suffolk, UK)

n/a, Not applicable

### Cell proliferation assay

#### WST-1 assay

Investigation of HOMEC proliferation was as previously described [8]. Briefly, cells were seeded at a density of 1 × 10^4^ cells per well in 2% (w/v) gelatin (Sigma, Poole, UK) coated 96-well plates (Greiner Bio One, Stonehouse, UK) and treated overnight in growth factor-deprived media containing 2% (v/v) FCS. After 24 h, treatments (1, 5, 10, 25, 50 and 125 ng/ml of Gall1 ± inhibitors) were added at the given concentrations (Table 1) and incubated for 48 or 72 h. Subsequently, WST-1 reagent (Roche, Welwyn Garden City, UK) was added to the assay medium and absorbance was measured at 450 nm against the blank in a PHERAstar BMG plate-reader.

#### BrdU assay

Cells were seeded in 2% gelatin pre-coated 96 well plates at a density of 20,000 cells/well in starvation media containing 2% FCS. After overnight incubation, cells were treated with or without Gal1 (1, 5, 25, 50 and 125 ng/ml) and incubated for 48 h. A commercially available BrdU reagent (Merck Chemicals Ltd., Nottingham, UK) was added to the wells for the last 24-h incubation and cellular proliferation was assessed (according to the manufacturer’s instructions) based on absorbance using a SpectraMax plate-reader (Molecular Devices, Berkshire, UK) set at dual wavelength of 450/550 nm.

#### CyQUANT assay

This procedure was performed according the manufacturers instruction. Briefly, after 48 h treatment with Gal1 (50 ng/ml), media was removed from each well, followed by addition of the dye binding solution (1X HBSS buffer) containing CyQUANT NF dye reagent (Fisher Scientific, Loughborough, UK). After 2-h incubation, the plates were read at Ex/Em: 485/530 using a FLUOstar BMG plate-reader (BMG Labtech Ltd, Bucks, UK) and cell proliferation was assessed based on the fluorescence intensity against the background containing HBSS buffer.

### HOMEC migration

Assessment of cellular migration was carried out using a Cultrex Cell 96 transwell migration assay (Bio-Techne Ltd., Abingdon, UK) as previously described (5). Briefly, cells were incubated in growth factor-deprived media supplemented with 0.5% FCS overnight. Next, cells were seeded at a density of 5 × 10^4^ in the upper assay chamber and treated ± Gal1 (50 ng/ml) and in the presence or absence of ERK1/2 and/or AKT inhibitors at their given concentrations (Table 1). Negative controls received carrier alone. After 6 h incubation at 37 °C, the bottom chambers were washed, followed by addition of cell dissociation solution/calcein AM for a further hour to label and detach migrated cells. Fluorescence in the bottom wells was read at Ex/Em: 485/520 nm.

### Human galectin-1 ELISA (Bio-Techne Ltd., Abingdon, UK)

HOMECs were treated ± CathL ± sulfasalazine (NFκB inhibitor, 100 µmol/l) for 4 min, 30 min, 2, 4, 8 and 24 h. Supernatants were collected, centrifuged at 200*g* for 10 min, and transferred into fresh microfuge tubes which were either stored at − 20 °C for future experiments or immediately used to detect levels of Gal1. The assay was carried out according to the manufacturer’s instructions.

### qRT-PCR

Total RNA from cultured HOMECs treated with CathL ± sulfasalazine was isolated with TRI reagent as per the manufacturer’s protocol (Sigma-Aldrich, Gillingham, UK). Extracted RNA was DNase-treated using a DNAfree kit (Ambion, Northumberland, UK). Complementary DNA (cDNA) was synthesised from 1 µg of RNA per sample using the qScript supermix kit (Quanta Biosciences, USA). Real-time RT-PCR was conducted using the TaqMan PrecisionPLUS master mix (Primer Design, Southampton, UK) with cycling conditions as follows: 1 cycle of 60 °C for 2 min and 95 °C for 2 min; 40 cycles of 95 °C for 10 s, 60 °C for 30 s; and 1 cooling cycle of 37 °C for 30 s, on a LightCycler 96 Real-time Detection System (Roche, Welwyn Garden City, UK). The specificity of the PCR product was confirmed by melting-curve analysis. The Gal1, and two house-keeping genes GAPDH and β-2-microtubulin primers were purchased from Thermo Fisher Scientific (Northumberland, UK). For each gene, crossing point (Cp) values were determined from the linear region of the amplification plot and normalized by subtraction of the geometric mean of the Cp values for two housekeeping genes. Relative expression was subsequently calculated using the 2-ΔCp approach, and the data were presented as fold change in LGALS1 gene expression (normalised to control).

### Patients

Female patients signed an informed consent form for the study which was reviewed by the Institutional Review Board (Gomel State Medical University), Gomel, Belarus. Sixty formalin-fixed and paraffin embedded archival tissue specimens including 20 cases of each of the following: normal omenta (from organ transplant donors), omenta of serous ovarian carcinoma patients without omental metastasis (SC wo MTS) and, with metastasis (SC w MTS), were used for immunohistochemistry (IHC). This was assessed by histopathologists D.A.Z. and S.L.A using the following criteria: a presence of papillary and solid pattern of tumour moderate to marked nuclear atypia, nuclear pleomorphism, stromal invasion, more than 12 mitotic figures in 10 high power fields [25]. Clinical information was obtained from the patients’ medical records. None of the patients within the control group had tumours or infections, or previous peritoneal surgical operations and traumas. The group of HGSC (SC wo MTS) patients were selected based on the following criteria: absence of secondary tumours, absence of previous chemotherapy treatment (intraoperative diagnostic of cancer), absence of previous peritoneal surgical operations and traumas, stages II–III (FIGO, 2009). The clinic-pathologic features of the ovarian serous carcinoma cases used in the present study are shown in Table 2. In stage II, cancer cells are found in other organs within the pelvis but not the omentum, and thus omenta from stage II patients were not included in the study [26].

**Table 2:** Clinic-pathologic characteristics of patients

Characteristics	SC wo MTS group (n = 20)	SC w MTS group (n = 20)
Age	49.1 (41.3–57.2)	48.5 (40.1–56.4)
FIGO stage		
IIA	11	–
IIB	9	–
IIIA	–	10
IIIB	–	10
Primary tumour grade		
Low grade	9	8
High grade	11	12
Ascites		
Absent	19	12
Present (> 100 ml)	1	8

#### Immunohistochemistry

Formalin-fixed, paraffin-embedded specimens were cut into 5 µm-thick sections and affixed to Thermo Super Frost slides (Thermo Fisher Scientific, Schwerte, Germany) and air-dried. This was followed by dewaxing in xylene and rehydration with descending graded alcohols to water. Tissue sections were immunostained using BenchMark XT (Roche-Ventana Medical Systems, Inc, Tucson, Arizona, USA). Heat-induced epitope retrieval was performed with Ventana Cell Conditioning 1 (Tris ethylenediaminetetraacetic acid buffer, pH 8.0) for 30 min on the instrument. The endogenous peroxidase activity was blocked with 3% hydrogen peroxide for 4 min. Slides were incubated with or without (negative control) primary antibodies (anti-Gal1, Abcam, Cambridge, UK; anti-CD34, Roche-Ventana, Arizona, USA) for 30 min at room temperature. The subsequent reactions were performed using components from UltraView DAB Detection Kit (Roche-Ventana, Arizona, USA), including incubation with universal (rabbit and mouse) secondary antibody (or 8 min, a biotin-free, horseradish-peroxidase enzyme-labeled polymer, and a signal visualization by 3,3′-diaminobenzidine hydrochloride. Sections were then counterstained with hematoxylin [27].

#### Analysis of immunohistochemistry staining

Morphometrical analysis was carried out using a Nikon Eclipse 50i (Nikon, Japan) and NIS-Elements software (Nikon, Japan). To evaluate Gal1 expression, each case was rated according to a score that added a scale of intensity of staining to the area of staining [19]. At least five high-power fields (HPFs) were chosen randomly, and endothelium of every microvessel was counted separately. The intensity of Gal1 staining was graded on the following scale: 0, no staining; 1+ , mild staining; 2+ , moderate staining; 3+ , intense staining.

To determine microvessel density as specified by Weidner, any CD34 brown staining endothelial cell or cell cluster that was clearly separated from adjacent microvessels, tumour cells and other connective tissue was considered a single countable microvessel [28]. Vessel lumens, although usually present, were not necessary for a structure to be defined as a microvessel, and red blood cells were not used to define a vessel lumen. Microvessels were determined blindly as the investigator was blinded to this variable. After selecting five microscopic fields of highest neovascularisation or hot spots, under low magnification, individual microvessels were counted manually and its area was counted using function “area” [29].

### Statistical analysis

Data are expressed as mean ± standard deviation (SD) and analysed using Mann–Whitney U test and one-way ANOVA test. A Spearman (r) test with Chadock scale were used for correlation analysis. The univariate and multivariate hazard ratios (HR) were estimated using Cox regression analysis. A *p* value of less than 0.05 was considered statistically significant. For all data, *n* represents the number of patients, wells or dishes tested under each condition and also the results from at least two primary cell populations. Statistical analysis was performed using SPSS Statistical Software Package (SPSS Inc, Chicago, USA) and GraphPad Prism (GraphPad Software, California).

## Results

### CathL induces secretion of Gal1

**Fig. 1:**
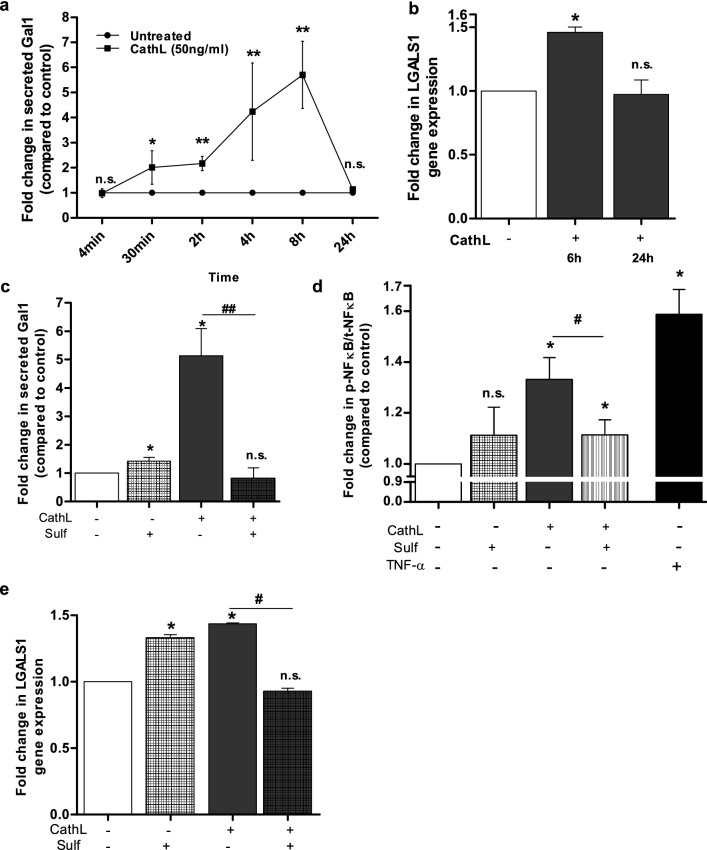
CathL-induced Gal1 secretion in HOMECs is transcriptionally regulated via activation of NFκB. **a** Cells were seeded and starved overnight in media supplemented with 2% FCS. Cells were then treated ± CathL (50 ng/ml) and supernatants were collected after 4 min, 30 min, 2, 4, 8 and 24 h treatment. A commercially available ELISA kit was used to assess the levels of extracellular Gal1 using a SpectraMax plate reader. Results are mean ± SD and represented as fold change in secreted Gal1 vs control. n = 4–6. **b** Cells were treated ± CathL (50 ng/ml) and lysed after 6 or 24 h treatment. Real-time PCR was performed on extracted RNA using a Roche LightCycler 96 and the data were normalised to GAPDH and β2 M. **c** Gal1 secretion was assessed with or without CathL ± sulfasalazine (100 µmol/l) for 8 h and the supernates were analysed as above. **d** CathL induces activation of NFκB (p65) in HOMECs. Cells were incubated with sulfasalazine (sulf, 100 µmol/l) or media alone for 24 h and then treated ± CathL (50 ng/ml) or positive control TNF-α (160 pg/ml) and in the absence or presence of sulfasalazine (100 µmol/l). After 4 h treatment, the phosphorylated level of NFkB was assessed using a commercially available cell-based ELISA kit. Results are mean ± SD and are represented as fold change in phosho-NFkB relative to total NFkB (compared to control). **e** CathL induces an upregulation of LGALS1 gene expression via NFkB activation. After overnight incubation and pre-incubation, cells were treated with CathL ± sulfasalazine for 6 h and analysed for gene expression as above. Results are mean ± SD and are represented as fold change in LGALS1 gene expression (relative to control), n = 4. *p < 0.05, **p < 0.01 vs control. #p < 0.05, ##p < 0.01 vs CathL. n.s. denotes not significant

Our recent data suggests a mitogen ligand-like activity for CathL in HOMECs [11], and our unpublished, preliminary data indicates that CathL induces a differential expression of galectin1 (Gal1) mRNA (LGALS1) in these cells. Therefore, we hypothesised that CathL induces production and secretion of Gal1. Figure 1a confirms that extracellular levels of Gal1 were, indeed, significantly increased in the presence of CathL between 30 min (twofold) and 8 h (5.2-fold) but returned to control levels after 24 h treatment (Fig. 1a). The latter may reflect Gal1 degradation since the protein has a reported serum half-life of 1.07 h [30] or re-uptake by the cells [31]. Interestingly, Gal1 release was not induced in HOMECs treated with the other HGSC-secreted factors insulin-like growth factor binding protein 7 and CathD (data not shown), suggesting that Gal1 secretion may be CathL-specific.

#### CathL induces upregulation of Gal1 mRNA expression

As stated above CathL rapidly (within 30 min) induced raised levels of extracellular Gal1, possibly reflecting secretion from intracellular stores or cleavage of cell surface Gal1 [32]. However, even greater levels of extracellular Gal1 were transiently detected after 4–8 h raising the possibility of additional transcriptional regulation (Fig. 1a). This was supported by the observation that 6 h CathL treatment also induced a transient, 1.5-fold, increase in LGALS1 mRNA level (p < 0.05) vs control (Fig. 1b) following a time course that coincided with the increase in extracellular Gal1 levels. These data suggested that CathL induced a transient increase in LGALS1 mRNA expression, Gal1 protein production and then secretion in HOMECs, and thus the upstream signalling pathway was investigated further.

### CathL induces LGALS1 expression via activation of NFκB

NFκB is a transcription factor involved in the production of many physiological and pathological proteins including Gal1 [22, 33] and thus, we examined the potential involvement of NFκB in CathL-induced Gal1 secretion in HOMECs. CathL-induced secretion of Gal1 was significantly reduced to control levels by sulfasalazine (inhibitor of NFκB) after 8 h treatment (0.9-fold vs 4.8-fold (CathL-treatment), both normalised to control; Fig. 1c). These data not only suggest that CathL induces NFkB activation (confirmed in Fig. 1d), but that CathL induces transcriptional regulation of Gal1 via NFκB activation. This was further supported by the qRT-PCR data demonstrating that sulfasalazine significantly reduced LGALS1 mRNA expression in CathL-treated cells after 6 h treatment (Fig. 1e). Interestingly, sulfasalazine alone increased LGALS1 mRNA levels in HOMECs (Fig. 1e). This could be due to a compensatory effect whereby other transcription factor(s), due to an inhibition of NFκB activity, may induce LGALS1 expression, which is also reflected in the protein level. These data suggest that CathL induces Gal1 secretion in HOMECs via activation of NFκB.

### Gal1 induces HOMEC proliferation via the ERK1/2 pathway, but not AKT

**Fig. 2:**
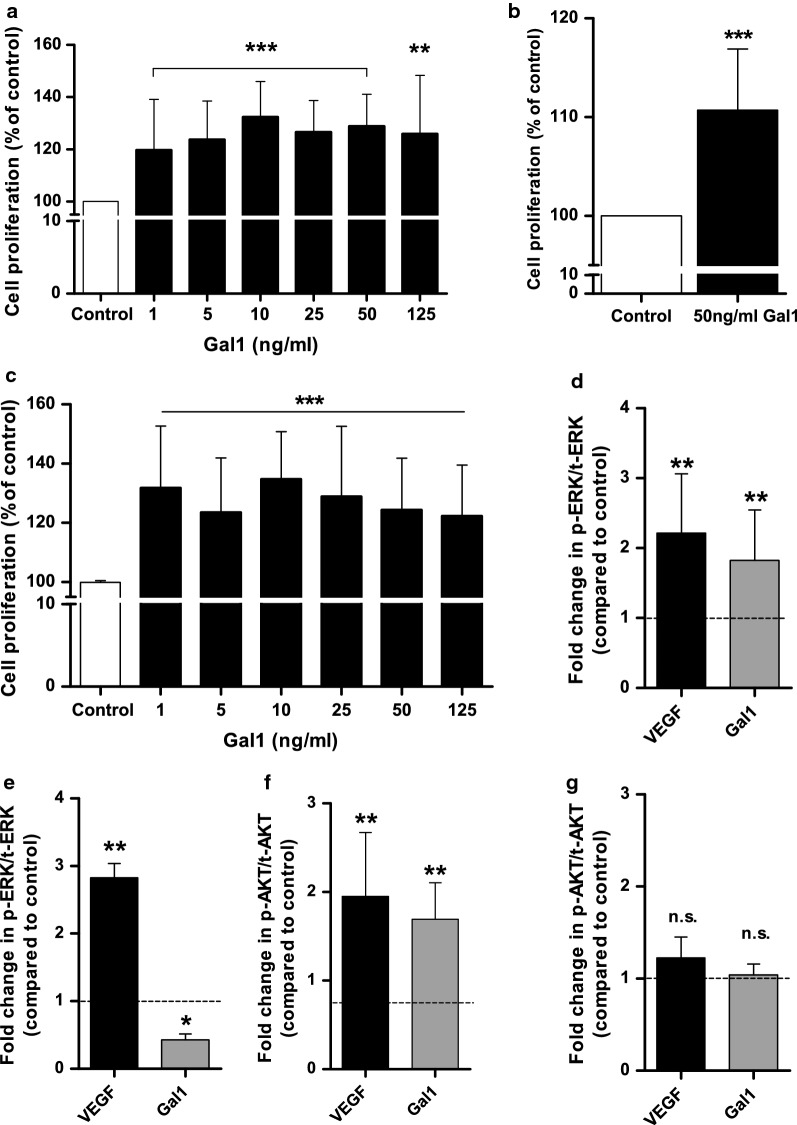
Gal1 induces HOMEC proliferation and activation of ERK1/2 and AKT. Cells were seeded in 2% gelatin pre-coated 96 well plates at a density of 10,000 cells/well in starvation media containing 2% FCS. **a**–**c** After overnight incubation, cells were treated ± Gal1 at various concentrations and incubated for 48 h. **a** WST-1 assay was used to assess cellular proliferation based on absorbance using a PHERAstar BMG plate-reader at 450 nm (n = 20). **b** Cell proliferation was examined at 50 ng/ml of Gal1 (CyQUANT). A commercially available CyQUANT reagent was used to assess cell proliferation after 48 h treatment based on fluorescence intensity using a FLUOstar BMG plate-reader at Ex/Em: 485/530 nm (n = 20). **c** A commercially available BrdU reagent was added to the wells for the last 24 h incubation and cellular proliferation was assessed (according to the manufacturer’s instructions) at 48 h based on absorbance using a SpectraMax plate-reader at 450/550 nm (n = 15). Results are mean ± SD and shown as percentage of the control, **p < 0.01 and ***p < 0.001 vs control (100%). After overnight incubation, cells were treated ± 50 ng/ml of Gal1 or 20 ng/ml of VEGF and incubated for 4 or 10 min. ERK1/2 (**d**, **e**) and AKT (**f**, **g**) phosphorylation was examined after 4 min (**d**, **f**) and 10 min (**e**, **g**) treatments. Commercially available cell-based ELISAs were used for the determination of ERK1/2 and AKT (S473) phosphorylation level. The ELISA experiments were carried out in quadruplets on two cell batches. The data show fold change in phosho-ERK1/2/AKT relative to total ERK1/2/AKT (compared to control). Results are mean ± SD, n.s., *p < 0.05, **p < 0.01 vs control (dotted lines); n = 4–6. n.s. denotes not significant vs control

We then examined whether the extracellular Gal1 released from HOMECs could have autocrine pro-angiogenic cellular effects i.e. on proliferation and migration. HOMEC proliferation was initially investigated over a range of Gal1 concentrations based on the concentrations of Gal1 (20–80 ng/ml) released into the supernatant of CathL-treated ECs over 8 h (data not shown), and the median range concentration (30–163 ng/ml) of Gal1 in the sera of HGSC stage I patients [19]. Since, we are investigating induction of angiogenesis in ECs from non-cancerous/non-metastatic omenta, we selected concentrations that fell within the lower spectrum of the in vivo range. Gal1 significantly induced cell proliferation at all concentrations tested vs control (100%, WST-1 assay) (Fig. 2a, Table 3). Additional studies with BrdU and CyQUANT proliferation assay kits confirmed that 50 ng/ml Gal1 significantly enhanced proliferation vs control (100%) (Fig. 2b, c). On the basis of these results and the levels of secreted Gal1, 50 ng/ml of Gal1 was selected for further experiments.

**Table 3:** Summary of the pro-proliferative effect of Gal1 on HOMECs at various concentrations (WST1 assay) (shown in Fig. 3a)

	Ctrl%	HOMEC proliferation (as % of control) at each Gal1 concentration (ng/ml)					
		1	5	10	25	50	125
48 h	100	119.7 ± 18.7	123.8 ± 14.8	132.3 ± 13.6	113.2 ± 13.7	128.9 ± 12.2	125.9 ± 22.4

HOMECs were ± increasing concentrations of Gal1 for 48 h. Results are mean ± SD and shown as percentage of control (100%). n = 11–20

The pro-proliferative role of Gal1 in HOMECs suggested possible activation of intracellular signalling pathways downstream of Gal1-cell surface interaction. We hypothesised activation of the ERK1/2 and PI3K/AKT pathways based on our previous publications [8, 11] in these cells. Gal1 treatment rapidly (< 4 min) increased phosphorylation of ERK1/2 (1.8-fold vs control, Fig. 2d), with phosphorylated levels decreasing to control levels after 10 min (Fig. 2e); suggesting a transient activation. Similar results were seen for AKT, with phosphorylation transiently increased (1.7-fold vs control; Fig. 2f, g) within 4 min of Gal1 treatment. The ELISA data were verified using specific inhibitors of ERK1/2 (U0126 and PD98059) and PI3K/AKT (LY294002 and MK2206) using previously determined concentrations (Additional file 1: Figure S1) [8, 11].

**Fig. 3:**
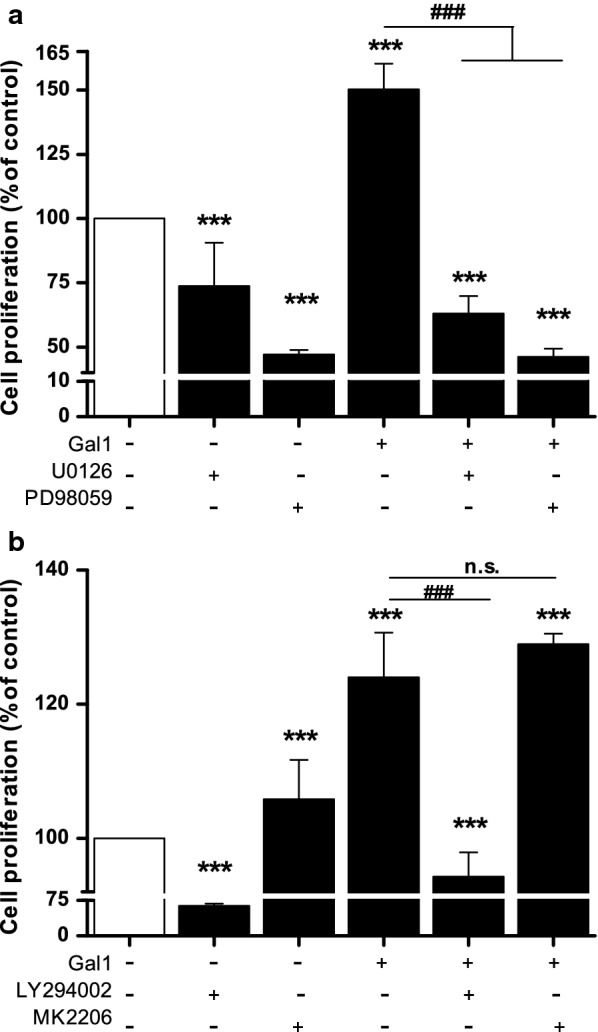
Gal1 induced HOMEC proliferation via the ERK1/2 and PI3K pathways, but not AKT pathway. Cells were seeded in 2% gelatin pre-coated 96 well plates at a density of 10,000 cells/well in starvation media containing 2% FCS. After overnight incubation, cells were treated ± Gal1 (50 ng/ml) and in the absence or presence of **a** U0126 (10 µmol/l) and PD98059 (25 µmol/L), **b** LY294002 (25 µmol/l) and MK2206 (5 µmol/l) and incubated for 72 h. Commercially available WST-1 assay was used to assess cellular proliferation. Results are mean ± SD and shown as percentage of the control, ***p < 0.001 vs control (100%), ###p < 0.001 vs Gal1 (normalised to control 100%), n = 14–15. n.s. denotes not significant

Since Gal1 induced activation of the ERK1/2 and PI3K/AKT pathways, we tested the effect of inhibition of these pathways on Gal1-induced HOMEC proliferation. Both ERK1/2 inhibitors, U0126 and PD98059, significantly reduced Gal1-induced cell proliferation (p < 0.001, Fig. 3a). Gal1-induced HOMEC proliferation was also significantly reduced by the PI3K inhibitor, LY294002 (p < 0.001) but the selective AKT inhibitor MK2206 had no effect (Fig. 3b, discussed later).

### Gal1 induces migration in HOMECs

**Fig. 4:**
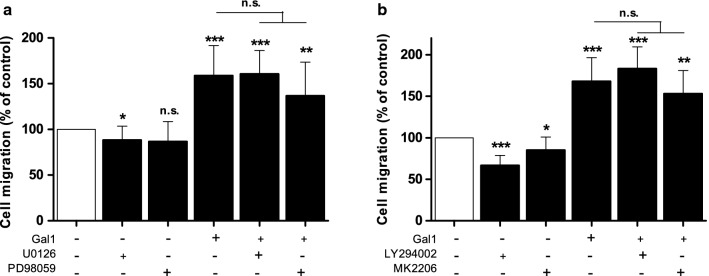
Gal1 does not induce HOMEC migration via the ERK1/2 or PI3K/AKT pathway. HOMECs were seeded in the upper transwell chamber and treated ± Gal1 (50 ng/ml) in the absence or presence of ERK1/2 inhibitors **a** U0126 (10 µmol/l) and PD98059 (25 µmol/l), or PI3K and AKT inhibitors **b** LY294002 (25 µmol/l) and MK2206 (5 µmol/l) respectively in media containing 0.5% FCS. The lower well contained corresponding treatments. After 6 h, migrated cells were stained with calcein AM and fluorescence was quantified by using a FLUOstar plate reader at Ex/Em: 485/520. Results are mean ± SD and shown as percentage of the control, *p < 0.05, **p < 0.01, ***p < 0.001 vs control (100%), n = 4–11. n.s. denotes not significant

A second key element of angiogenesis is EC migration and thus, the pro-migratory effect of Gal1 and the pathways involved were examined using inhibitors of ERK1/2 and AKT as above. Interestingly, although Gal1 significantly induced HOMEC migration (163.4 ± 47.2% vs control, 100%) none of the inhibitors tested significantly reduced this (Fig. 4a, b). These data combined with the ELISA data (Additional file 1: Figure S1) suggest that Gal1-induced HOMEC migration may not solely depend on the activation of the ERK1/2 and AKT pathways, and that other kinases may be involved.

### High positive correlation between in vivo Gal1 expression and number and area of vessels—a potential proangiogenic role for Gal1 in HOMECs

The in vitro data presented above suggest that HOMEC-secreted Gal1 may play a role in pro-angiogenic changes in the omental microvasculature. To examine this further in human disease we immunohistochemically investigated expression of Gal1 protein levels in the microvascular endothelium in patient samples; specifically, in normal omenta (control), omenta with HGSC metastasis (SC w MTS) and omenta with no metastatic tumour (SC wo MTS).

**Fig. 5:**
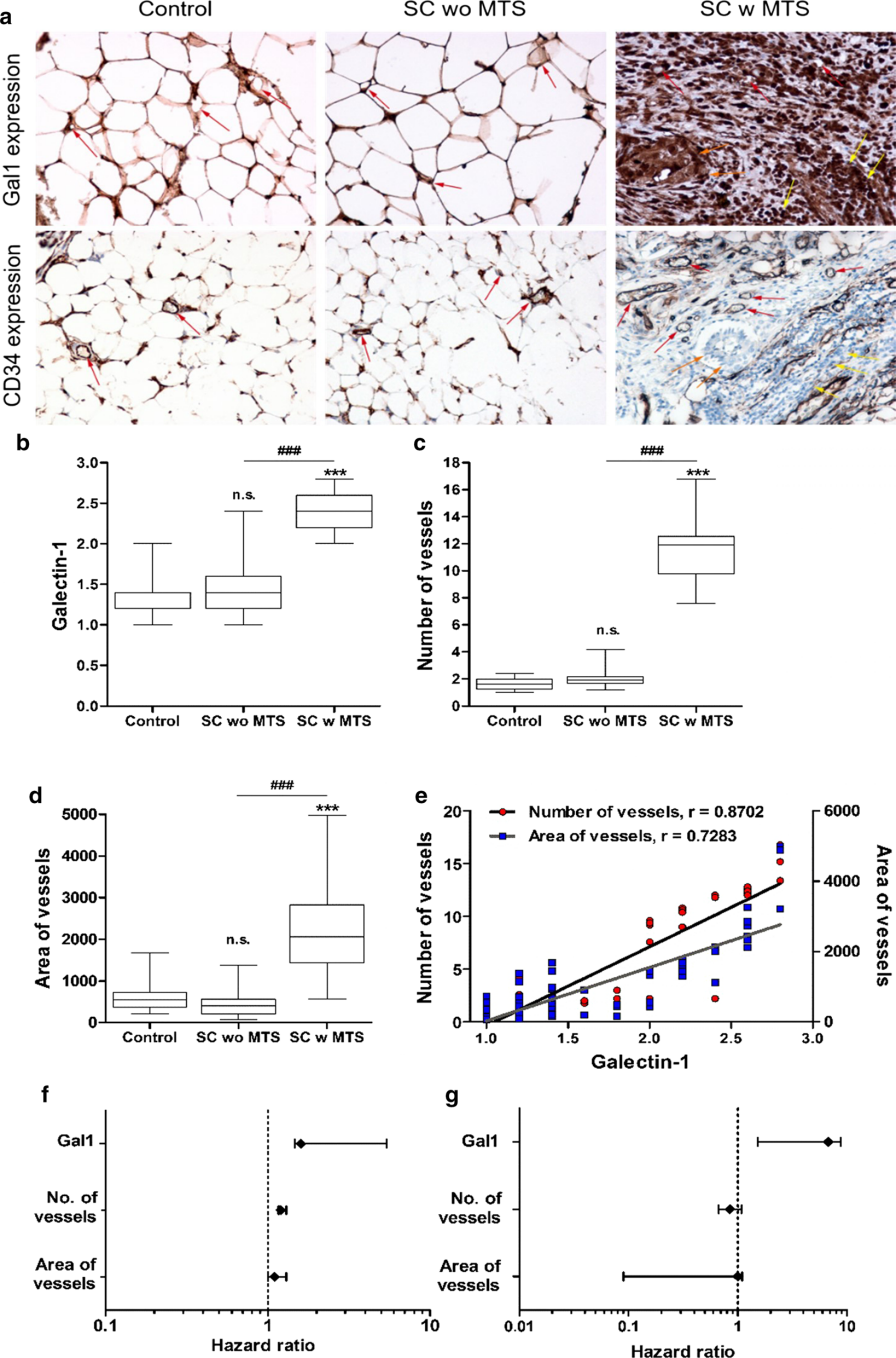
High positive correlation between Gal1 expression and number of vessels, and area of vessels. **a** Expression of Gal1 (upper panel) and CD34 (lower panel) in endothelium (moderate expression- red arrows) in control (left), SC wo MTS (serous carcinoma without omental metastasis, moderate expression; middle), and SC w MTS (serous carcinoma with omental metastasis, high expression; right). In SC w MTS, Gal1 expression is shown in endothelium (red arrows), cancer cells (orange arrows) and stromal lymphocytes infiltration (yellow arrows). **b** Significantly higher Gal1 expression in SC w MTS group compared to control and SC wo MTS. Gal1 expression is represented as an intensity score, as described by Chen et al. [19]. **c** A significant increase in the number of vessels and **d** area of vessels were found in SC w MTS group compared to the other groups. The vessels were counted according to Weidner’s method described in “Materials and method” section [28]. **e** Very high correlation between Gal1 and number, and area of vessels, indicating a potential proangiogenic role for Gal1. **f** Univariate and **g** multivariate hazard ratios of Cox analysis of predictive patients’ survival. Photographs were taken using a Nikon Eclipse 50i. ***p < 0.001 vs control group, ###p < 0.001 vs SC wo MTS. n.s. denotes not significant. Magnification: ×200

Interestingly, although the microvessels stained positively for Gal1 in all groups (Fig. 5a), the intensity of Gal1 expression (designated 1–3, see supplementary methods and [19]) differed between the groups. In the control, SC wo MTS and SC w MTS groups, Gal1 mean scores were 1.32 ± 0.29, 1.42 ± 0.37 and 2.39 ± 0.28 respectively with statistically significant differences (p < 0.001) between the control and the SC w MTS group, and the SC wo MTS and SC w MTS groups (Fig. 5b).

Additionally, we investigated microvessel density (as assessed by CD34 staining of the endothelium), a measure of vascularisation i.e. angiogenesis, in each group (Fig. 5a). There was a significant increase (p < 0.001) in vessel density in the SC w MTS group compared with both the control and SC wo MTS groups (Fig. 5c), confirming a significant increase in microvessel density in the omenta invaded by tumour. No non-specific staining was observed in our negative controls of each group (Additional file 2: Figure S2).

We also measured area of vessels (per 1 mm^2^) and found a significant increase in microvessel area (p < 0.001) in the SC w MTS group compared with SC w/o MTS and control groups (Fig. 5d).

An association study revealed a positive correlation between Gal1 and number of vessels (Spearman coefficient, r = 0.8702, p < 0.001), and area of vessels (r = 0.7283, p < 0.001) (Fig. 5e), suggesting a potential proangiogenic role for Gal1 in the omentum of advanced ovarian carcinoma.

Univariate Cox regression analysis of prognosis factors showed a significant association between predictive patient survival and Gal1 expression (p < 0.001) and number of vessels (p = 0.032), but not area of vessels (Fig. 5f); possibly due to atypical vessels—a phenomenon seen in tumour invasion. Interestingly, in multivariate Cox regression analysis only Gal1 expression (p < 0.001) (but not area or number of vessels) demonstrated significant prognostic value in ovarian cancer patients (Fig. 5g), suggesting that Gal1 expression could be a prognostic factor in advanced ovarian cancer with a role in angiogenesis. These data all strongly support a proangiogenic role for Gal1 in HGSC metastasis to the omentum.

## Discussion

Treatment of HGSC remains challenging due to late diagnosis and limited effective therapeutic strategies for advanced metastatic disease. This is compounded by the lack of understanding of the molecular mechanisms involved in the establishment and growth of the metastases; in particular the angiogenic activators that transform the local, host microvascular endothelium into a pro-angiogenic state. Targeting this process is an attractive therapeutic strategy. Indeed, previous therapies have concentrated on disruption of the VEGF pro-angiogenic pathway e.g. bevacizumab. However, these strategies have had disappointing results [3–7], highlighting the need to identify new potential targets for HGSC e.g. alternative proangiogenic factors.

One of the major sites of HGSC metastasis is the omentum and, we have previously reported that omental angiogenesis during metastasis of HGSC may occur independently of the VEGF/VEGFR axis. Specifically, we showed that CathD and CathL, two HGSC-secreted enzymes, significantly induced proangiogenic endothelial responses in omental ECs (HOMECs) [8, 11]. Here we show that CathL may induce secretion of Gal1 from HOMECs which acts as an autocrine pro-angiogenic factor in the local microvasculature, contributing to secondary tumour growth. One important aspect of these studies is the use of omental microvascular ECs. Since ECs display heterogeneity in their proteomics, morphology and functionality, it is essential to undertake studies using cells from a relevant vascular bed.

Gal1 has been shown to be involved in tumour progression, development and angiogenesis [21, 23]. However, a role for CathL-induced extracellular Gal1 in HGSC metastasis is a novel finding. The observation that CathL-induced increases in extracellular Gal1 levels were detected within 30 min suggests release from cytoplasmic stores [12, 32]. The enhanced CathL-induced cellular release of Gal1 at 4 and 8 h suggested an additional transcriptional regulation of LGALS1 which was confirmed by increased expression of LGALS1 with CathL treatment. Although CathL has not previously been reported to induce expression of LGALS1 or secretion of Gal1, transcriptional regulation of Gal1 has been reported in ECs, specifically, LDL-induced expression of Gal1 in human aortic ECs [34].

CathL significantly enhanced NFkB phosphorylation and the NFkB inhibitor, sulfasalazine, significantly reduced this phosphorylation as well as LGALS1 expression and Gal1 secretion, supporting a role for NFkB in CathL-induced Gal1 secretion. We reported previously that CathL acts non-proteolytically on these cells and induces proangiogenic changes. Thus, it is possible that CathL binds to an, as yet, unidentified receptor and induces this pathway.

**Fig. 6:**
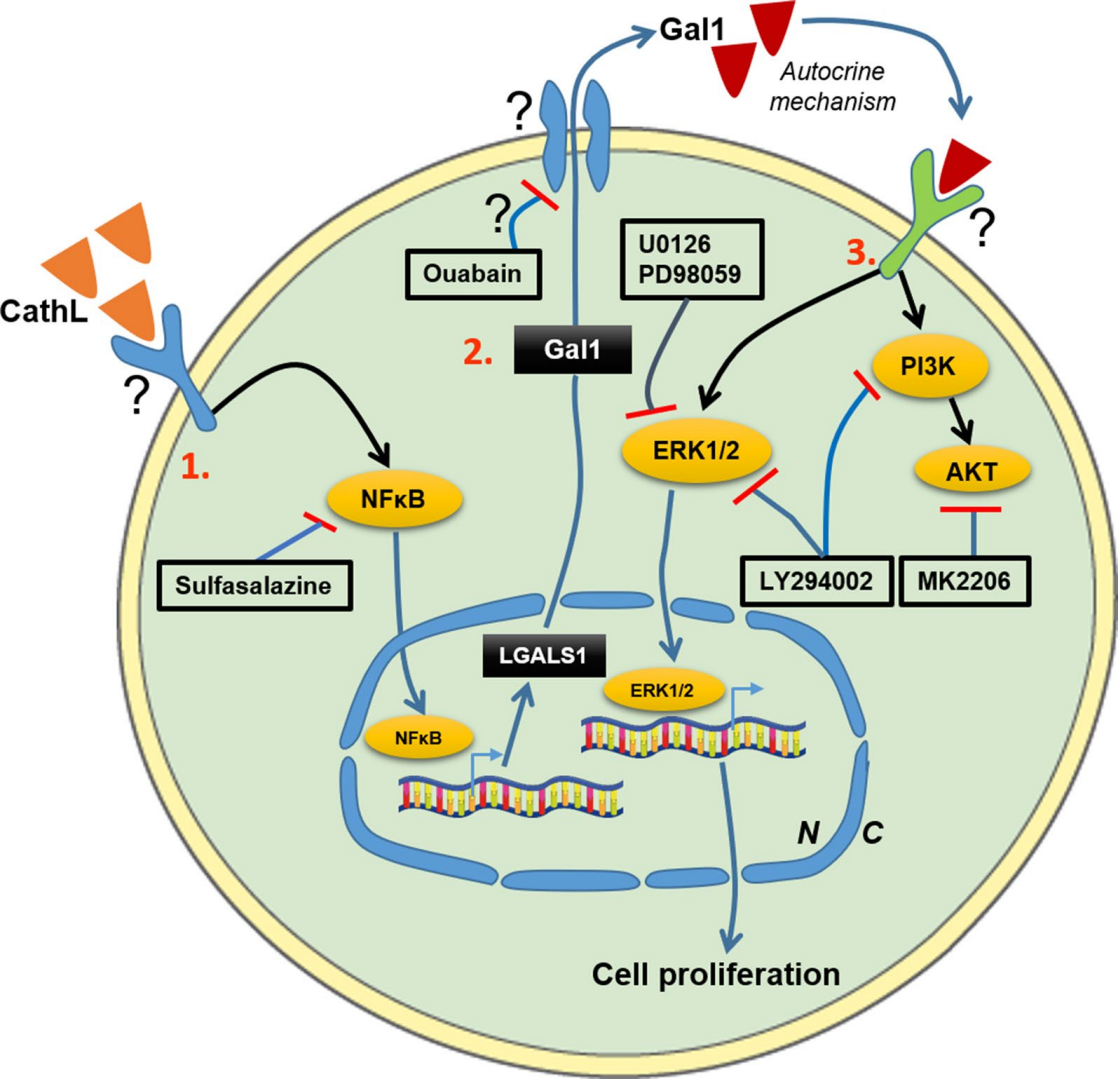
A summary of the proposed CathL-induced Gal1 secretion pathway and its pro-proliferative function and potential therapeutic targets in HOMECS. 1. CathL non-proteolytically activates a cell surface receptor, possibly a receptor tyrosine kinase, which leads to an increase in activated levels of the transcription factor NFκB, resulting in increased expression of LGALS1 (Gal1 mRNA). Sulfasalazine, an inhibitor of NFκB, reduces LGALS1 expression and subsequent secreted level of Gal1. 2. LGALS1 is transcribed and translated into Gal1 which is secreted out of the cell, possibly utilising a Na^+^/K^+^ ATP-pump, via a non-conventional secretory pathway. 3. Secreted Gal1 induces activation of MAP kinase ERK1/2 and PI3K/AKT via an autocrine mechanism and an unknown receptor(s), where the ERK1/2 pathway induces HOMEC proliferation. The MEK/ERK1/2 inhibitors U0126 and PD98059 significantly reduce this cellular function by inhibiting ERK1/2 phosphorylation. Both the PI3K inhibitor LY294002 and AKT inhibitor MK2206 inhibit phosphorylation of AKT at Ser473 (S473) in Gal1-treated HOMECs. However, only LY294002, but not MK2206, inhibits Gal1-induced HOMEC proliferation, suggesting a cross-reactivity of PI3K inhibitor LY294002 with the ERK1/2 pathway. Potential therapeutic targets are: Gal1 secretory mechanism, Gal1 binding to its receptor(s), and blocking receptor(s) for Gal1. N, nucleus; C, cytoplasm

Next, we showed that Gal1 acted as a potent pro-angiogenic factor in HOMECs, stimulating both proliferation and migration; an observation that agrees with other findings showing pro-proliferative effects of exogenous Gal1 in human ECs [23, 31]. We then investigated the potential signalling pathways activated downstream by Gal1; concentrating on two key signalling molecules ERK1/2 and AKT. Gal1 induced significant and transient phosphorylation of both, an effect which was abolished by inhibitors of MEK1/2 (upstream of ERK1/2, U0126 and PD98059) and PI3K/AKT (LY294002 and MK2206). Gal1-induced HOMEC proliferation was significantly reduced by the two MEK1/2 inhibitors and the PI3K inhibitor LY294002, but not the selective AKT inhibitor MK2206. It is possible that the latter result was observed due to cross-reactivity of LY294002 with the ERK1/2 pathway and thus inhibition of ERK1/2 phosphorylation [35]. Taken together, our data suggest that ERK1/2 pathway is involved in the HOMEC proliferation induced by exogenous Gal1 (Fig. 6).

Gal1 also had a potent pro-migratory effect on HOMECs. However, this was not impacted by the ERK1/2 or AKT inhibitors despite previous reports that these kinases are involved in cell migration [8, 11]. In ECs Thijssen et al. [31] previously suggested involvement of activated ERK1/2 in Gal1-induced HUVEC migration, however Hsieh et al. [36], reported that activated JNK, a MAP kinase, is responsible for Gal1-induced migration in HUVECs, highlighting the potential involvement of other kinases in HOMEC migration.

We then carried out immunohistochemical analysis of patient samples to complement the in vitro studies. Although adipocytes are known to express Gal1 on their cell membrane [37], strikingly, there was a significant increase in Gal1 expression in the microvessel endothelium and surrounding stromal cells in the omenta with metastatic serous carcinoma compared with normal or non-metastatic serous carcinoma omenta, with a strong positive correlation between Gal1 staining intensity and both microvessel number and vessel area. A significant Gal1 expression and its role in angiogenesis and metastasis has also been reported in cancer-associated stroma in primary ovarian tumour, breast and pancreatic cancers [38–40]. Here, we show high-intensity Gal1 expression in cancer-associated stroma in secondary lesions for the first time in the metastasised omenta. These data, support the possibility that Gal1 is a potent proangiogenic factor in HGSC. Since we previously showed increased endothelial expression of CathL in the omenta of patients with metastasised serous carcinoma [41], the current observation of a similar increased Gal1 expression pattern in comparable patient samples could signify a physiological role for CathL induction of Gal1 expression in angiogenesis in this pro-metastatic disease.

We also demonstrated a significant association between increased Gal1 expression and a poor predictive survival for HGSC patients. Interestingly, Schulz and colleagues reported that overexpression of Gal1 was associated with increased migratory and invasive behaviour, and decreased sensitivity to cisplatin, in HGSC cells; possibly explaining the decreased survival of HGSC patients with increased Gal1 expression [42].

In summary, our data suggest that the HGSC-secreted factor CathL induces secretion of Gal1 from HOMECs which, via an autocrine mechanism, stimulates pro-angiogenic cellular responses i.e. proliferation and migration in the omental vasculature during metastasis of HGSC.

Our observation that Gal1 may play an important pro-angiogenic role is supported by previous reports indicating a role for Gal1 in tumour progression, partly by triggering angiogenesis [20]. For instance, in vitro, elevated Gal1 induced HUVECs to migrate and proliferate and in in vivo *studies,* Gal1 knockout mice had severely compromised tumour growth and vessel density [21]. In a variety of cancers including prostate, colon and thyroid [17], Gal1 is highly expressed in cancer-associated stroma, and this over-expression correlates with pathological factors including advanced disease stage, tumour invasion and increased rates of disease reoccurrence [19]. Over-expression of Gal1 has also been found to increase tumour cell invasion in the OVCAR-3 cell line [19]. Moreover, when the serum levels of Gal1 were compared in ovarian cancer patients alongside the most widely used biomarker CA125, it was found that around 70% of patients were correctly identified as positive by both proteins [19], suggesting that Gal1 could potentially be used as a biomarker for HGSC progression, as well as outcome.

## Conclusions

Identification of new targets is a priority in advanced HGSC. Our data indicate a strong proangiogenic effect of Gal1 on omental ECs, with high positive correlation between Gal1 and microvessel density in vivo, highlighting that the Gal1 pathway could be a potential therapeutic target in HGSC metastasis to the omentum. Although anginex, a non-specific anti-angiogenic compound, has been shown to reduce angiogenesis by binding and possibly blocking cell membrane-bound Gal1 [21, 43], there is currently no FDA-approved therapy in place to block potent effects of secreted Gal1. However, there are a number of potential Gal1-related cellular targets [44] (Fig. 6), for instance, the secretory pathway of Gal1 and potential Gal1-binding receptors that could potentially be targeted in the quest to find an effective treatment for metastasised HGSC.

## Additional files

**Additional file 1: Figure S1.** Inhibitors of ERK1/2 and AKT reduce ERK1/2 and AKT phosphorylation respectively in intact HOMECs. After overnight incubation in starvation media (containing 2% FCS), cells were pre-incubated with the ERK1/2 inhibitors a) U0126 (10 μmol/l) and PD98059 (25 μmol/l) or PI3K/AKT inhibitors b) LY294002 (25 μmol/l) and c) MK2206 (5 μmol/l) for (a) 20–30 min or (b+ c) 2.5 h, and then co-treated with or without 50 ng/ml of Gal1 or 20 ng/ml of VEGF for 4 min. Commercially available cell-based ELISAs were used for determination of phosphorylation levels. The data show fold change in phospho-protein relative to total protein (compared to control). Results are mean ± SD, *p<0.05, **p<0.01 vs control (1-fold, dotted lines), #p<0.05 vs VEGF/Gal1 (normalised to control), n = 4.

**Additional file 2: Figure S2.** Tissue sections were stained only with secondary antibody to both Gal1 and CD34 in control group, groups with serous carcinoma without metastasis (SC wo MTS) and with metastasis (SC w MTS). Photographs were taken using a Nikon Eclipse 50i. Magnification ×200.

## Data Availability

The research materials supporting this publication can be accessed by contacting Jacqueline Whatmore (J.L.Whatmore@exeter.ac.uk).

## Abbreviations

AKT(S473): protein kinase B
CathD: cathepsin D
CathL: cathepsin L
cDNA: complimentary DNA
EC: endothelial cell
EOC: epithelial ovarian cancer
ERK1/2: extracellular signal-regulated kinases 1/2
FCS: foetal calf serum
Gal1: galectin-1
HOMECs: human omental microvascular endothelial cells
HPF: high-power field
HUVECs: human umbilical vein endothelial cells
JNK: c-Jun N-terminal kinases
LGALS1: galectin-1 mRNA
MAP: mitogen-activated protein kinase
MEK: mitogen-activated protein kinase kinase
NFkB: nuclear factor kappa-light-chain-enhancer of activated B cell
PBMCs: peripheral blood mononuclear cells
SC w MTS: serous carcinoma with metastasis
SC wo MTS: serous carcinoma without metastasis
TNF-a: tumour necrosis factor-alpha
VEGF: vascular endothelial growth factor
