# Supplementary figures and images for: Cathepsin L-induced galectin-1 may act as a proangiogenic factor in the metastasis of high-grade serous carcinoma
Source: J Transl Med. 2019 Jul 3;17:216. doi: 10.1186/s12967-019-1963-7 (PMC6610868; doi:10.1186/s12967-019-1963-7)

a)

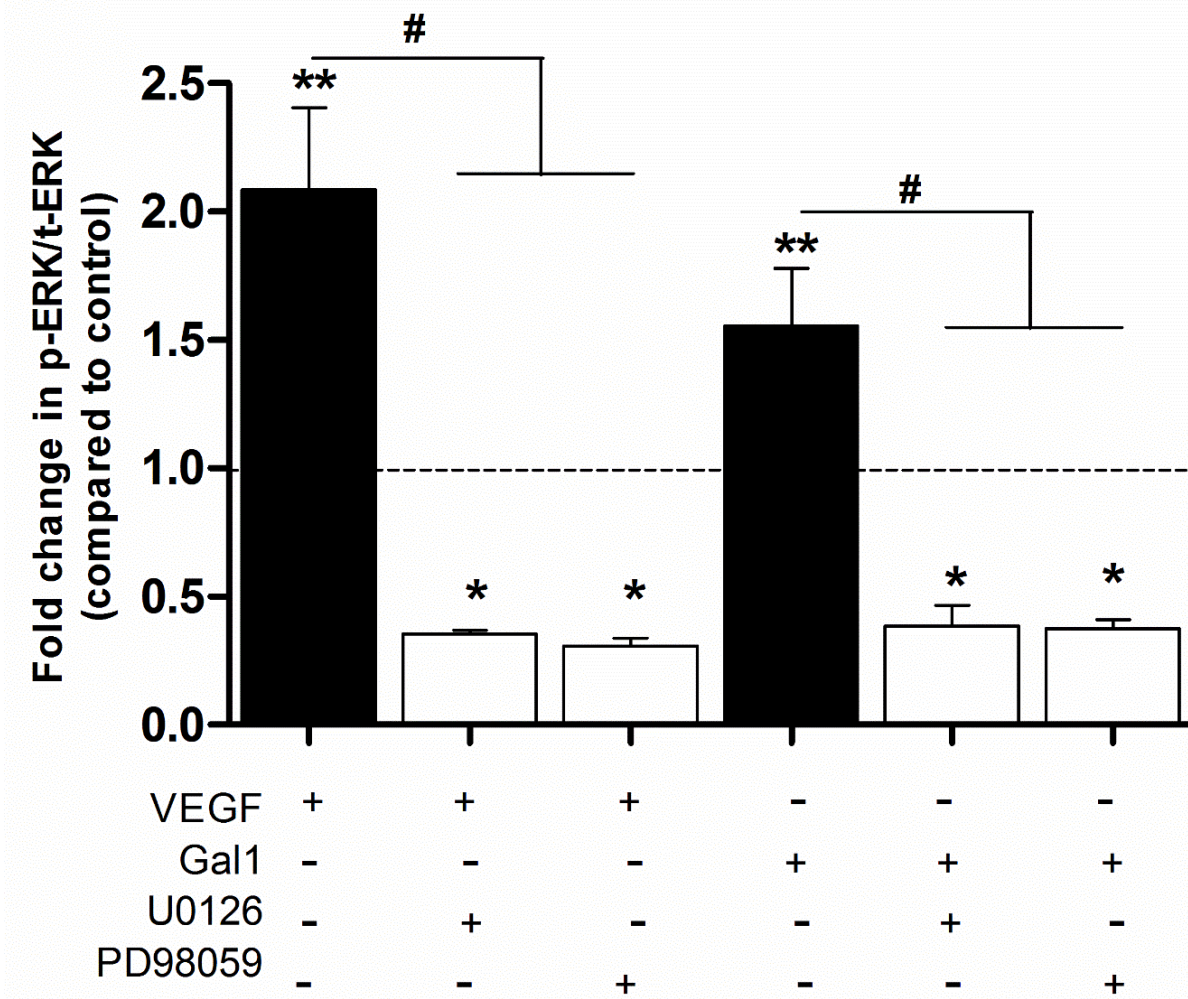

b)

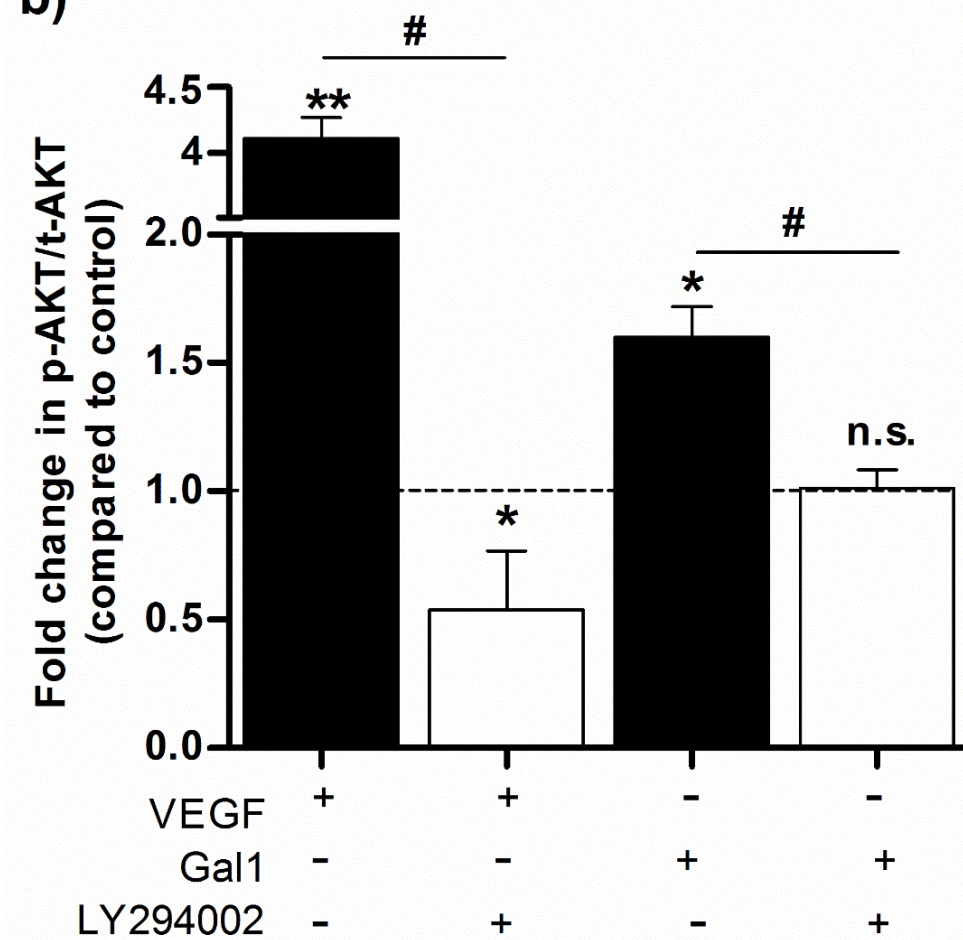

c)

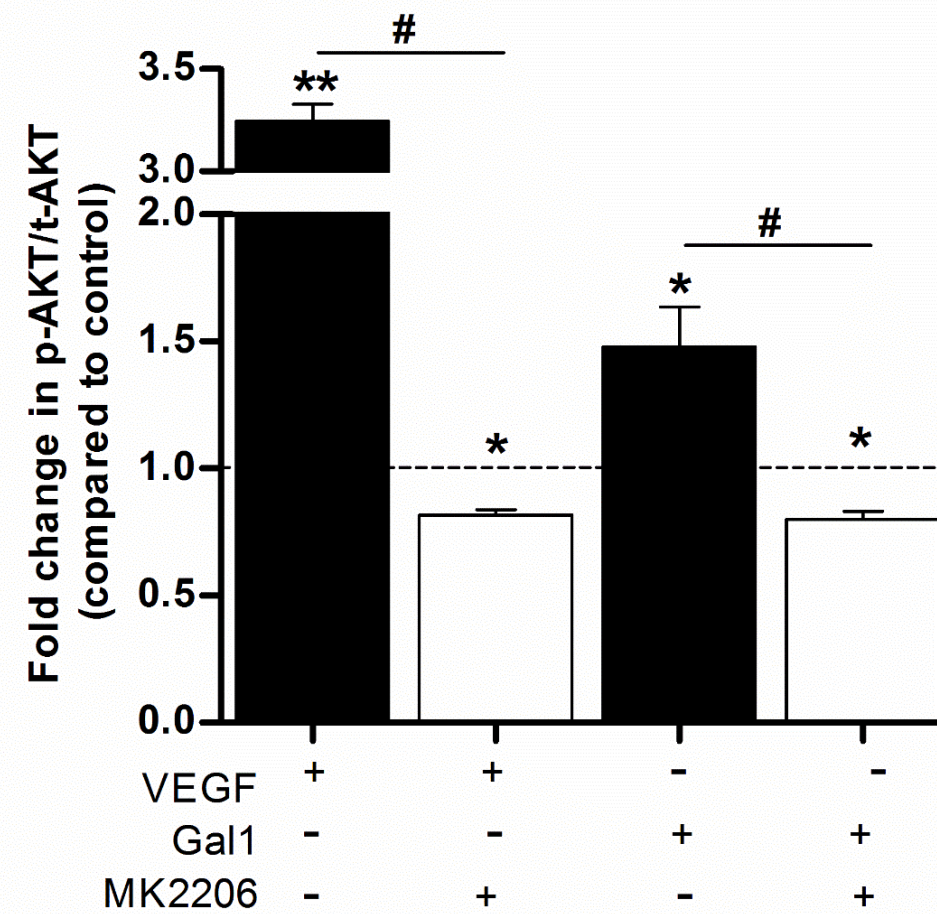

Supplement: Supplementary file 1 — Additional file 1: Figure S1. Inhibitors of ERK1/2 and AKT reduce ERK1/2 and AKT phosphorylation respectively in intact HOMECs. After overnight incubation in starvation media (containing 2% FCS), cells were pre-incubated with the ERK1/2 inhibitors a) U0126 (10 μmol/l) and PD98059 (25 μmol/l) or PI3K/AKT inhibitors b) LY294002 (25 μmol/l) and c) MK2206 (5 μmol/l) for (a) 20–30 min or (b+ c) 2.5 h, and then co-treated with or without 50 ng/ml of Gal1 or 20 ng/ml of VEGF for 4 min. Commercially available cell-based ELISAs were used for determination of phosphorylation levels. The data show fold change in phospho-protein relative to total protein (compared to control). Results are mean ± SD, *p<0.05, **p<0.01 vs control (1-fold, dotted lines), #p<0.05 vs VEGF/Gal1 (normalised to control), n = 4. [file 12967_2019_1963_MOESM1_ESM.pdf]

Control

SC wo MTS

SC w MTS

Negative controls

Gal1

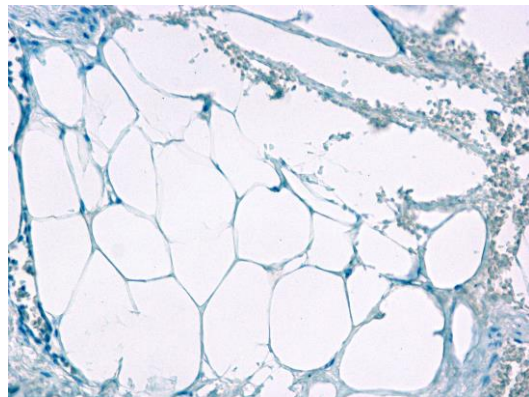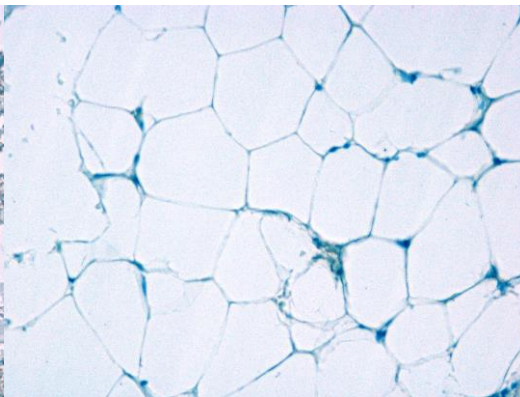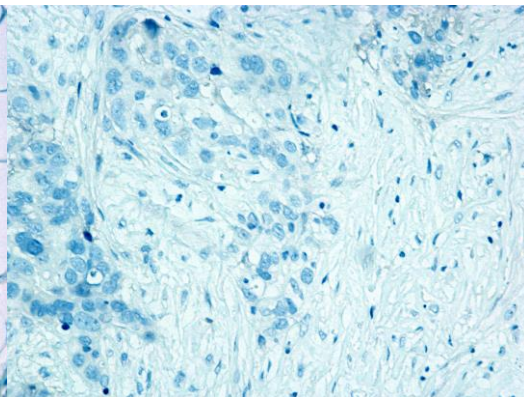

CD34

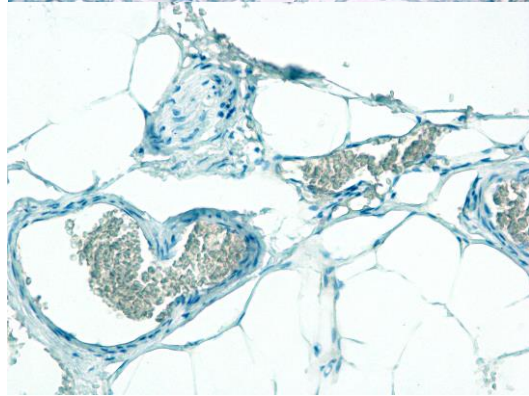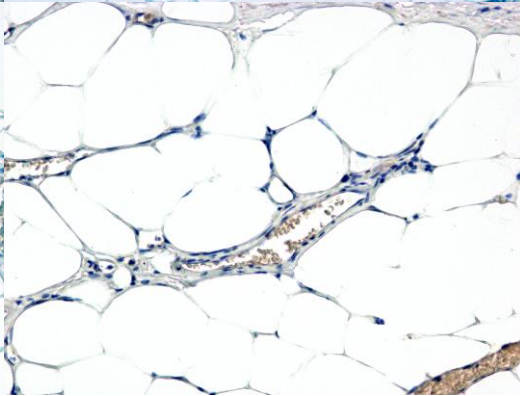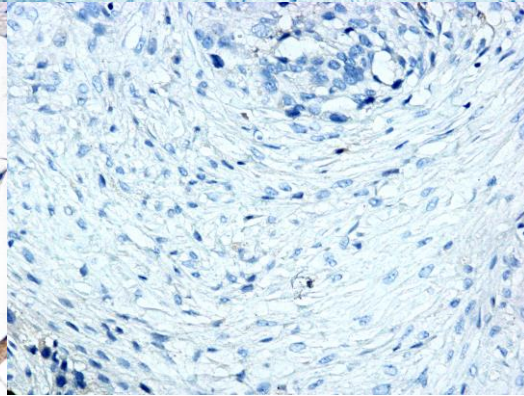

Supplement: Supplementary file 2 — Additional file 2: Figure S2. Tissue sections were stained only with secondary antibody to both Gal1 and CD34 in control group, groups with serous carcinoma without metastasis (SC wo MTS) and with metastasis (SC w MTS). Photographs were taken using a Nikon Eclipse 50i. Magnification ×200. [file 12967_2019_1963_MOESM2_ESM.pdf]
